# Ectopic *TLX1* Expression Accelerates Malignancies in Mice Deficient in *DNA-PK*

**DOI:** 10.1371/journal.pone.0089649

**Published:** 2014-02-26

**Authors:** Konstantin Krutikov, Yanzhen Zheng, Alden Chesney, Xiaoyong Huang, Andrea K. Vaags, Valentina Evdokimova, Margaret R. Hough, Edwin Chen

**Affiliations:** 1 Institute of Medical Science, University of Toronto, Toronto, Ontario, Canada; 2 Department of Molecular and Cellular Biology, Sunnybrook Research Institute, Toronto, Ontario, Canada; 3 Department of Clinical Pathology, Sunnybrook Health Sciences Centre, Toronto, Ontario, Canada; 4 Department of Laboratory Medicine and Pathobiology, University of Toronto, Toronto, Ontario, Canada; Wayne State University, United States of America

## Abstract

The noncluster homeobox gene *HOX11/TLX1* (*TLX1*) is detected at the breakpoint of the t(10;14)(q24;q11) chromosome translocation in patients with T cell acute lymphoblastic leukemia (T-ALL). This translocation results in the inappropriate expression of *TLX1* in T cells. The oncogenic potential of *TLX1* was demonstrated in *IgHμ-TLX1^Tg^* mice which develop mature B cell lymphoma after a long latency period, suggesting the requirement of additional mutations to initiate malignancy. To determine whether dysregulation of genes involved in the DNA damage response contributed to tumor progression, we crossed *IgHμ-TLX1^Tg^* mice with mice deficient in the DNA repair enzyme DNA-PK (*Prkdc^Scid/Scid^* mice). *IgHµ-TLX1^Tg^Prkdc^Scid/Scid^* mice developed T-ALL and acute myeloid leukemia (AML) with reduced latency relative to control *Prkdc^Scid/Scid^* mice. Further analysis of thymi from premalignant mice revealed greater thymic cellularity concomitant with increased thymocyte proliferation and decreased apoptotic index. Moreover, premalignant and malignant thymocytes exhibited impaired spindle checkpoint function, in association with aneuploid karyotypes. Gene expression profiling of premalignant *IgHµ-TLX1^Tg^Prkdc^Scid/Scid^* thymocytes revealed dysregulated expression of cell cycle, apoptotic and mitotic spindle checkpoint genes in double negative 2 (DN2) and DN3 stage thymocytes. Collectively, these findings reveal a novel synergy between TLX1 and impaired DNA repair pathway in leukemogenesis.

## Introduction

The homeobox gene *TLX1* encodes a member of the nonclustered subclass of homeodomain-containing transcription factors. *TLX1* was initially identified at the breakpoint of the t(10;14)(q24;q11) reciprocal chromosome translocation in patients with T cell acute lymphoblastic leukemia (T-ALL) [1], [2], [3]. This translocation places the entire *TLX1* coding region under the transcriptional control of the T cell receptor δ (*TCRδ*) promoter resulting in inappropriate expression of *TLX1* in T cells, and is found in ∼5% of pediatric T-ALL and 30% of adult T-ALL cases. *TLX1*-initiated T-ALL exhibits a block at the stage of β-selection [4]. This maturation arrest might be due to down regulation of the *BCL11B* tumor suppressor gene [5], [6] or as a result of recruitment of TLX1 by the transcription factor ETS1 to the enhancer of the TCR-α locus leading to repression of Vα-Jα rearrangements [7]. *TLX1* is not typically expressed in adult tissues but is critical for the development of the spleen during embryogenesis [8], [9], [10].

Transgenic mice have played a central role in defining the molecular mechanisms of *TLX1*-initiated hematopoietic malignancies. Transgenic mice in which the *TLX1* gene is placed under the control of the immunoglobulin heavy chain (*IgH*) promoter and enhancer, thus directing its expression predominantly to the B cell compartment (*IgHµ-TLX1^Tg^* mice), develop mature marginal zone B-cell lymphomas after an extended latency [11]. Surprisingly, although *IgHµ-TLX1^Tg^* mice express elevated levels of *TLX1* in thymocytes during the early stages of T lymphopoiesis, no cases of T-ALL have been detected in these mice. More recently, p56*^Lck^*-*TLX1* and doxycyline repressible *tet-TLX1* transgenic mice with T-cell specific *TLX1* expression have been reported [5], [12]. *TLX1* expression in T cells induces a block in thymocyte development at the DN2 stage and, similar to human *TLX1*-initiated T-ALL, *TLX1* transgenic mice develop cortical stage tumors with a heterogeneous CD4 and CD8 immunophenotype. Additionally, the doxycyline repressible *tet-TLX1* transgenic mice subsequently acquire activating *NOTCH1* mutations, consistent with reports that more than 50% of T-ALL patients carry *NOTCH1* activating mutations [13] and that NOTCH1 and TLX1 can coregulate transcription in T-ALL cells [14].

An association of TLX1 with aberrant cell cycle checkpoint regulation has been known for many years. In the first such report, TLX1 was shown to interact with protein phosphatases PP2A and PP1 to disrupt a G2/M cell cycle checkpoint [15]. Subsequently, high throughput comparison of gene expression profiles of two cell lines established from patients with *TLX1*-positive T-ALLs revealed modulated expression of numerous genes associated with G1/S progression [16]. More recently, multiple groups reported that ectopic expression of TLX1 results in dysregulation of the spindle checkpoint [5], [17], attributable, in part, to down-regulated expression of the mitotic checkpoint regulator CHEK1 [5]. One possible manifestation of such widespread subversion of cell cycle checkpoint control would be a loss of genomic integrity. Indeed, *TLX1*-transgenic mice exhibit a heightened predisposition for the development of whole chromosome copy changes [5], [17], [18].

*Prkdc* encodes the catalytic subunit of the DNA-dependent protein kinase (DNA-PKcs) which plays a critical role in the non-homologous end joining (NHEJ) pathway of DNA repair. It is also critical for V(D)J recombination, a process which relies on NHEJ to promote immune system diversity at the *Ig* or *TCR* loci. Mice harboring homozygous germline inactivating mutations in the *Prkdc* gene (*Prkdc^Scid/Scid^*) are viable but have severe combined immunodeficiency due to an inability to undergo V(D)J recombination. B cell development in these mice is arrested at the B220^+^CD19^+^IgM^-^ stage, whereas thymocyte development is arrested at the DN3 stage [19]. Critically, DNA-PK deficiency in mice contributes to genomic instability [20], [21], [22], [23] and *Prkdc^Scid/Scid^* mice are prone to the spontaneous development of T-ALL, AML and nonthymic tumors [24], [25], [26].

In this report, we tested whether increased genomic instability associated with DNA-PK loss collaborates with *TLX1* to accelerate T-ALL. To this end, we generated double mutant *IgHμ-TLX1* ^Tg^ *Prkdc^Scid/Scid^* mice and showed that these mice exhibit statistically significant accelerated onset of leukemia relative to *Prkdc^Scid/Scid^* mice. Moreover, we identify numerous genetic pathways that are perturbed in association with *TLX1* overexpression, including those involved in chromosome segregation, cell cycle checkpoints and apoptosis.

## Results

### Similar Premalignant Phenotypes in Young *IgHμ-TLX1^Tg^Prkdc^Scid/Scid^* and *Prkdc^Scid/Scid^* Mice

To determine whether dysregulation of a DNA repair pathway collaborated with ectopic expression of *TLX1* in disease progression, we crossed *IgHμ-TLX1^Tg^* mice with CB17 ICR-*Prkdc^scid^* mice (referred to as *Prkdc^Scid/Scid^* mice). We chose to use the ICR background as it most resembles the outbred background of the CD1-TLX1 mice.

The thymi and spleens of control *Prkdc^Scid/Scid^* mice and double mutant *IgHμ-TLX1^Tg^Prkdc^Scid/Scid^* mice were initially examined at 6 weeks of age, prior to the development of any overt leukemia. Thymi from *IgHμ-TLX1^Tg^Prkdc^Scid/Scid^* mice exhibited significantly reduced cellularity compared to wildtype mice, but was increased relative to those of *Prkdc^Scid/Scid^* mice (Table 1). Spleens of *IgHμ-TLX1^Tg^Prkdc^Scid/Scid^* mice were also reduced in cellularity as compared to wild type mice, but were also increased as compared to spleens of *Prkdc^Scid/Scid^* littermates (Table 1). Due to the lack of lymphoid cells, Peyer’s patches and lymph nodes of *IgHμ-TLX1^Tg^Prkdc^Scid/Scid^* and *Prkdc^Scid/Scid^* mice were small and difficult to detect. Histologically, thymi of *IgHμ-TLX1^Tg^Prkdc^Scid/Scid^* and *Prkdc^Scid/Scid^* mice lacked cortico-medullary delineation and consisted of predominantly epithelial cells and immature thymocytes. In the spleen, lymphoid follicles and germinal centers were devoid of lymphoid cells and populated with fibroblasts and plasma cells. Bone marrow histology appeared normal.

**Table 1:** Absolute thymocyte and splenocyte numbers in premalignant mice.

	Wild type (×10^6^ cells) n = 20	*Prkdc^Scid/Scid^* (×10^6^ cells) n = 20	*IgHμ-TLX1^Tg^Prkdc^Scid/Scid^* (×10^6^ cells) n = 20
**Thymocytes**	151±60	0.69±0.12	1.52±0.32
**Splenocytes**	122±60	8.13±2.8	10.26±3.2

### Expression of the *TLX1* Transgene in Thymocytes of *IgHμ-TLX1^Tg^Prkdc^Scid/Scid^* Mice

**Figure 1:**
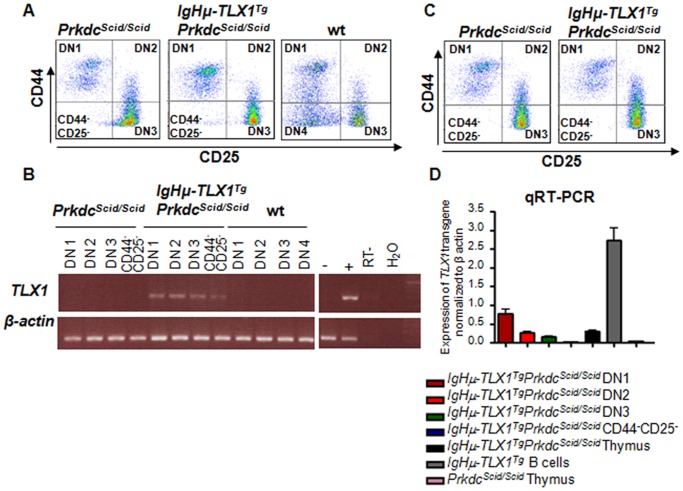
Expression of *TLX1* in *IgHμ-TLX1^Tg^Prkdc^Scid/Scid^* premalignant thymocytes. (A) OP9-DL1 co-cultures were initiated using fetal liver-derived *IgHµ-TLX1^Tg^Prkdc^Scid/Scid^, Prkdc^Scid/Scid^* and wild type (WT) HSCs. Thymocytes were harvested on day 7 and flow sorted into DN1, DN2, DN3 and CD44^-^CD25^-^ fractions on the basis of CD25 and CD44 expression. (B) RT-PCR analysis showing expression of the *TLX1* transgene in *IgHµ-TLX1^Tg^Prkdc^Scid/Scid^, Prkdc^Scid/Scid^* and WT thymocytes expanded in the OP9-DL1 co-culture system. Purified B220^+^ splenocytes isolated from wild type and *IgHµ-TLX1^Tg^* mice were used as negative and positive controls, respectively. *TLX1* expression was not detected in immature thymocytes derived from *Prkdc^Scid/Scid^* and WT mice. (C) Total thymocytes were obtained from thymi of six week old *IgHµ-TLX1^Tg^Prkdc^Scid/Scid^* and *Prkdc^Scid/Scid^* mice and DN1, DN2, DN3 and CD44^-^CD25^-^ fractions were flow sorted based on CD44 and CD25 expression. (D) qRT-PCR analysis showing expression of the *TLX1* transgene in DN1, DN2, DN3 and CD44^-^CD25^-^ thymocytes flow sorted from the thymi of *IgHµ-TLX1^Tg^Prkdc^Scid/Scid^* and *Prkdc^Scid/Scid^* mice. B220^+^ splenocytes from healthy *IgHµ-TLX1^Tg^* mice were used as a positive control whereas thymocytes from *Prkdc^Scid/Scid^* mice were used as a negative control. Expression of the *TLX1* transgene in total thymocytes of *IgHµ-TLX1^Tg^Prkdc^Scid/Scid^* and *Prkdc^Scid/Scid^* mice is also shown.

The *TLX1* transgene in the double mutant mice was driven by the *IgHμ* promoter. Although the activity of the *IgHμ* promoter is typically restricted to B cells, leaky expression in thymocytes and myeloid progenitors has been reported [27], [28]. Therefore, to quantitate *IgHμ*-directed *TLX1* expression during T lymphopoiesis, thymocytes were derived from wild type, *Prkdc^Scid/Scid^* and *IgHμ-TLX1^Tg^Prkdc^Scid/Scid^* mice by co-culture of murine fetal liver cells-derived hematopoietic stem cells (HSCs) on OP9-DL1 stromal cells and *TLX1* expression was analyzed by RT-PCR (Figure 1A). Weak *TLX1* transgene expression was detected in DN1, DN2 and DN3 thymocytes and in CD44^-^CD25^-^ cells (Figure 1B). Expression was down regulated as cells progressed from DN1 to the DN3 stage and was significantly decreased in CD44^-^CD25^-^ cells, consistent with declining activity of the *IgHµ* promoter during thymocyte maturation as previously reported [27], [28]. Expression of *TLX1* was not detected in DN1, DN2, DN3 or CD44^-^CD25^-^ thymocytes derived from *Prkdc^Scid/Scid^* or wild type mice indicating that the *TLX1* detected originated from the *IgHμ-TLX1* transgene and not the endogenous mouse *Tlx1* gene. To confirm that the *TLX1* transgene was also expressed *in vivo*, quantitative RT-PCR analysis of thymocytes obtained from six week old *IgHμ-TLX1^Tg^Prkdc^Scid/Scid^* and *Prkdc^Scid/Scid^* age- and sex-matched littermates was performed (Figure 1C). Expression of the *TLX1* transgene was detected in DN1, DN2, DN3 and CD44^-^CD25^-^ thymocytes from *IgHμ-TLX1^Tg^Prkdc^Scid/Scid^* mice (Figure 1D). Of note, the level of expression of the *IgHμ-TLX1* transgene in thymocytes was significantly lower than that detected in purified B220^+^ splenocytes from *IgHμ-TLX1^Tg^* mice.

### Accelerated Leukemogenesis in *IgHμ-TLX1^Tg^Prkdc^Scid/Scid^* Mice

**Figure 2:**
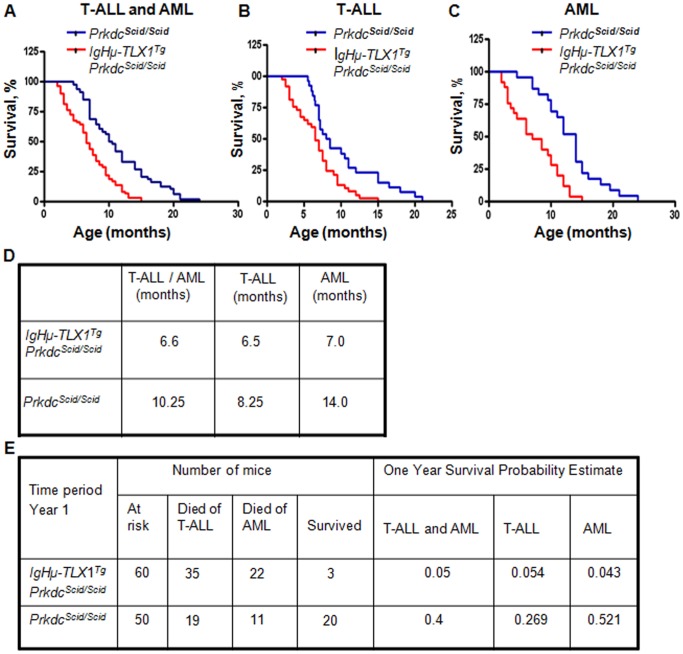
*TLX1* accelerates T-ALL and AML in *IgHµ-TLX1^Tg^Prkdc^Scid/Scid^* mice. Cohorts of mice were monitored for signs of disease for 25 months. A diagnosis of AML or T-ALL was made based on histological examination of bone marrow, spleen and thymic tissues. Survival of *Prkdc^Scid/Scid^* and *IgHµ-TLX1^Tg^Prkdc^Scid/Scid^* mice are indicated by blue or red lines, respectively. (A) Disease free survival of *Prkdc^Scid/Scid^* and *IgHµ-TLX1^Tg^Prkdc^Scid/Scid^* mice during the observation period, (p<0.0001). (B) T-ALL free survival of *Prkdc^Scid/Scid^* and *IgHµ-TLX1^Tg^Prkdc^Scid/Scid^* mice during the 25 month observation period, (p<0.003). (C) AML free survival of *Prkdc^Scid/Scid^* and *IgHµ-TLX1^Tg^Prkdc^Scid/Scid^* mice during the 25 month observation period, (p<0.0001). (D) Median survival of the complete *IgHµ-TLX1^Tg^Prkdc^Scid/Scid^* and *Prkdc^Scid/Scid^* mouse cohorts developing T-ALL and/or AML. The column labeled as T-ALL/AML corresponds to Figure 1A and shows the median survival of *IgHµ-TLX1^Tg^Prkdc^Scid/Scid^* and *Prkdc^Scid/Scid^* mice from cohorts developing either T-ALL or AML. The columns labeled T-ALL and AML correspond to Figure 1B and 1C and indicate median survival of *IgHµ-TLX1^Tg^Prkdc^Scid/Scid^* or *Prkdc^Scid/Scid^* mice from cohorts developing T-ALL or AML respectively. (E) A one-year Kaplan-Meier survival probability estimate of the complete cohort of *IgHµ-TLX1^Tg^Prkdc^Scid/Scid^* and *Prkdc^Scid/Scid^* mice developing T-ALL or AML.

To determine whether ectopic expression of *TLX1* collaborated with DNA-PK deficiency in the pathogenesis of hematopoietic malignancies, a cohort of 60 *IgHμ-TLX1^Tg^Prkdc^Scid/Scid^* mice and 50 age and sex matched *Prkdc^Scid/Scid^* mice were followed for 24 months. At the end of this period, all mice had developed a malignancy with histological features and flow cytometric profiles consistent with either T-ALL or acute myeloid leukemia (AML) (Figure 2A). However, *IgHμ-TLX1^Tg^Prkdc^Scid/Scid^* mice succumbed to disease at earlier time points relative to *Prkdc^Scid/Scid^* littermates. The median survival of the complete cohort of *IgHµ-TLX1^Tg^Prkdc^Scid/Scid^* and *Prkdc^Scid/Scid^* mice, irrespective of disease diagnosis, was 6.6 and 10.25 months, respectively (Figure 2D). The one-year survival probability estimate of *IgHµ-TLX1^Tg^Prkdc^Scid/Scid^* mice was also decreased relative to those of *Prkdc^Scid/Scid^* littermates developing either T-ALL or AML (Figure 2E).

**Figure 3:**
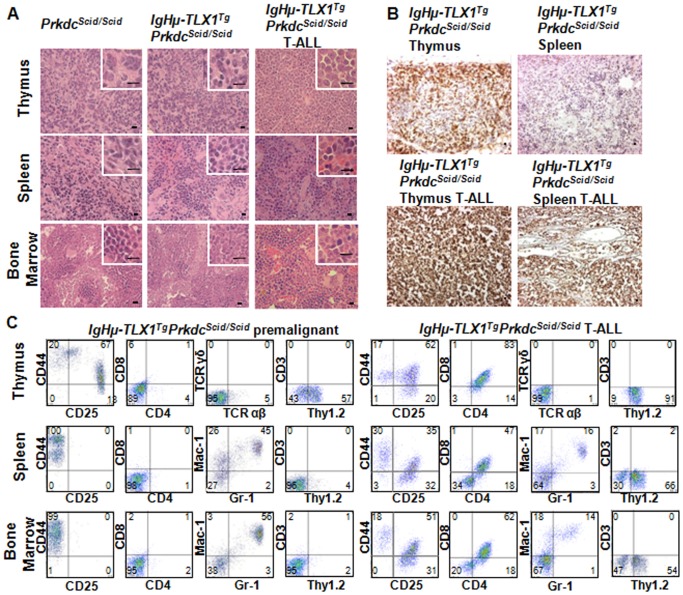
*TLX1-*induced T-ALL in *IgHµ-TLX1^Tg^Prkdc^Scid/Scid^* mice. (A) Hematoxylin and eosin staining of tissues isolated from premalignant *Prkdc^Scid/Scid^* and *IgHµ-TLX1^Tg^Prkdc^Scid/Scid^* mice and *IgHµ-TLX1^Tg^Prkdc^Scid/Scid^* mice diagnosed with T-ALL. Magnification x40 (overview) and x100 (insert). Scale bars, 10 µm. (B) Immunohistochemical analysis of thymus and spleen from a premalignant *IgHµ-TLX1^Tg^Prkdc^Scid/Scid^* mouse and a moribund *IgHµ-TLX1^Tg^Prkdc^Scid/Scid^* mouse stained with an anti-Thy1.2 antibody. Magnification x20. Scale bars, 10 µm. (C) Cells from thymi, spleens and bone marrow of premalignant and moribund *IgHµ*-*TLX1^Tg^Prkdc^Scid/Scid^* mice were examined for cell surface expression of CD44, CD25, CD4, CD8, CD3, TCRαβ, TCRγδ and Thy1.2 (for T cells) and Gr-1 and Mac-1 (for myeloid cells).

In total, 59% of *IgHμ-TLX1^Tg^Prkdc^Scid/Scid^* mice and 54% of *Prkdc^Scid/Scid^* mice developed an immature T-cell leukemia. The median survival of *IgHμ-TLX1^Tg^Prkdc^Scid/Scid^* mice with T-ALL was 6.5 months, whereas *Prkdc^Scid/Scid^* mice exhibited a protracted latency with a median survival of 8.25 months (Figure 2B, D). Histological examination of tissues of *Prkdc^Scid/Scid^* and *IgHµ-TLX1^Tg^Prkdc^Scid/Scid^* mice exhibiting T-ALL indicated thymic involvement in 100% of animals, while splenic and bone marrow infiltration of tumor cells was detected in 67% and 52% of mice, respectively. This suggests that the thymus was the primary site for disease initiation. The architecture of the thymus was disrupted with indistinguishable cortico-medullary delineation. The spleens had disrupted follicles, with patchy areas infiltrated with a large, relatively uniform population of lymphocytes having polylobulated pleomorphic nuclei with diffuse, loose, speckled, open chromatin and prominent nucleoli (Figure 3A).

Immunohistological analysis was performed using frozen sections of thymi and spleens isolated from age- and sex-matched healthy and terminal *IgHμ-TLX1^Tg^Prkdc^Scid/Scid^* mice. Staining of sections with the pan T-cell antibody, Thy1.2, revealed numerous Thy1.2^+^ cells in the subcortical area of the thymi while Thy1.2^+^ cells were absent in the medullar area of thymi and were not detected in the spleens of healthy mice. In contrast, a homogeneous population of Thy1.2^+^ tumor cells was detected throughout the thymi and spleens of terminal mice (Figure 3B).

**Figure 4:**
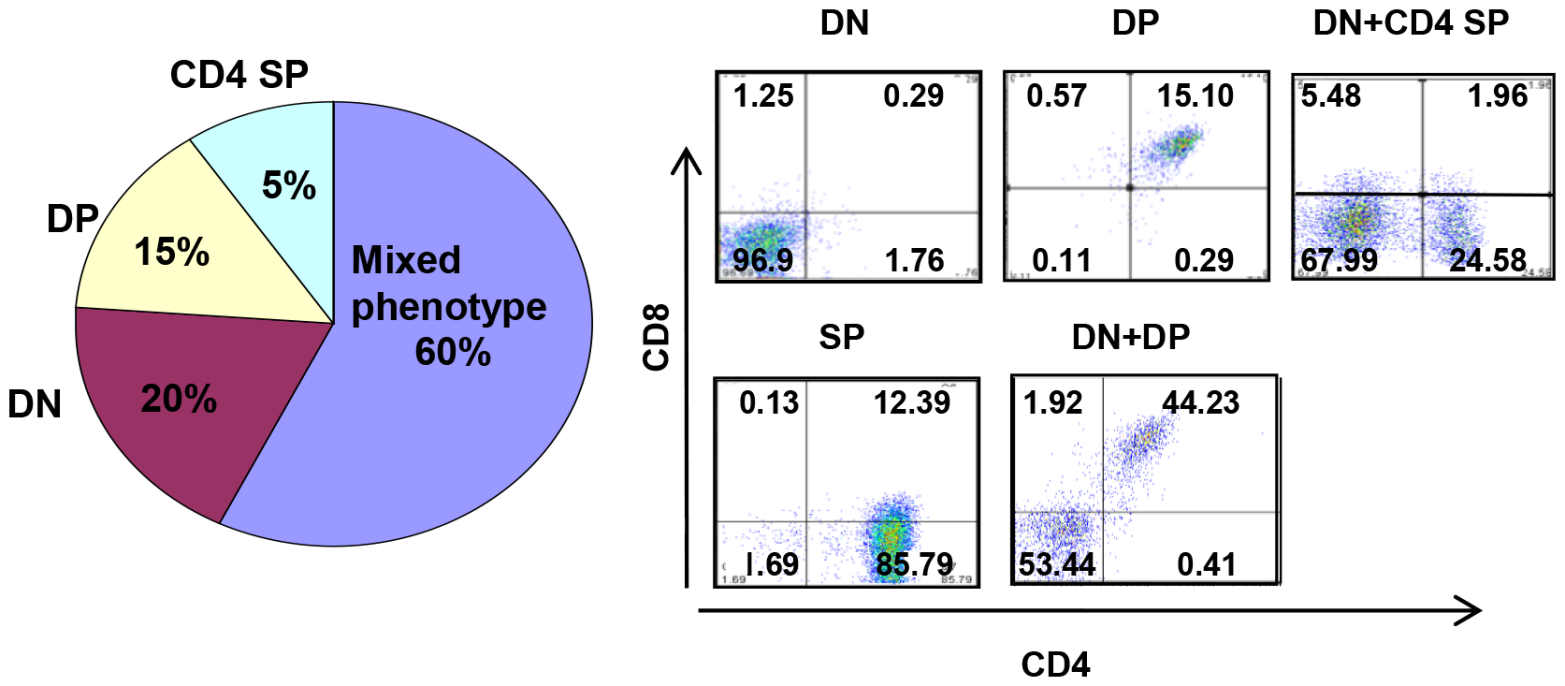
Immunophenotype of *TLX1*-induced T-ALL. Immunophenotype distribution showing heterogeneous expression of CD44, CD25, CD4 and CD8 in *IgHµ-TLX1^Tg^Prkdc^Scid/Scid^* T-ALL. Representative flow diagrams showing heterogeneous expression of CD4 and CD8 in *TLX1*-initiated leukemia are presented.

Flow cytometric analysis revealed heterogeneity in the tumor phenotype with respect to expression of CD4 and CD8 (Figure 3C) with tumor cells isolated from the thymus, spleen and bone marrow expressing both CD4 and CD8 or only CD4 with decreased levels of expression of CD44 and CD25. Other tumors consisted of CD44 and CD25 expressing cells with profiles typical of DN1, DN2 and DN3 cells or only DN3 thymocytes. Tumor cells did not expressed αβ- or γδ- T cell receptors, indicative of an immature thymocyte phenotype (Figure 3C). The majority (60%) of thymic tumors had a mixed phenotype expressing low-levels of CD4 and CD8 along with a combination of CD44 and CD25 (Figure 4).

**Figure 5:**
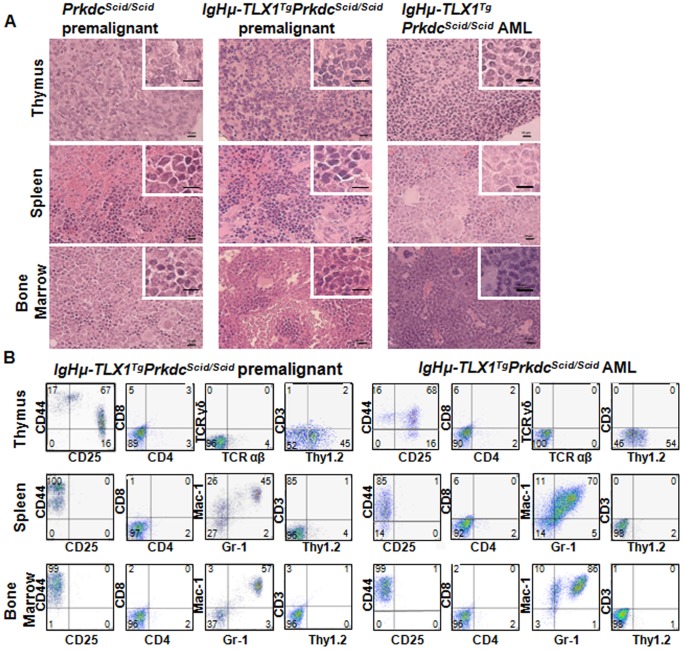
*TLX1-*induced AML in *IgHµ-TLX1^Tg^Prkdc^Scid/Scid^* mice. (A) Hematoxylin and eosin staining of tissues isolated from premalignant *Prkdc^Scid/Scid^* and *IgHµ-TLX1^Tg^Prkdc^Scid/Scid^* mice and from *IgHµ-TLX1^Tg^Prkdc^Scid/Scid^* mice diagnosed with AML. Magnification x40 (overview) and x100 (insert). Scale bars, 10 µm. (B) Cells from thymi, spleens and bone marrow of premalignant and moribund *IgHµ-TLX1^Tg^Prkdc^Scid/Scid^* mice were examined for cell surface expression of CD44, CD25, CD4, CD8, CD3, TCRαβ, TCRγδ and Thy1.2 (for T cells) and Gr-1 and Mac-1 (for myeloid cells).

Forty one percent of *IgHμ-TLX1^Tg^Prkdc^Scid/Scid^* mice developed AML, with a median survival of 7.0 months and 46% of *Prkdc^Scid/Scid^* control mice developed AML with a median survival of 14.0 months (Figure 2C, D). In these cases, histological analysis of bone marrow and spleens from terminally ill mice revealed the presence of leukemic cells with a primitive myeloid phenotype (Figure 5A). Histological analysis indicated the absence of tumor cells in the thymus while flow cytometry analysis using CD44 and CD25 antibodies indicated that thymi contained a normal profile of immature DN thymocytes typical for mice with the *Prkdc^Scid/Scid^* mutation (Figure 5B). Moreover, thymocytes did not express TCRβ, CD3, CD4 and CD8 receptors and thus, had not progressed beyond the DN3 stage of development. Flow cytometric analysis of bone marrow and spleens cells stained with Gr-1 and Mac-1 antibodies revealed an increased proportion of Mac-1^lo^Gr-1^lo^, Mac-1^hi^Gr-1^int^ and Mac-1^hi^Gr-1^lo^ cells in *IgHμ-TLX1^Tg^Prkdc^Scid/Scid^* mice relative to *Prkdc^Scid/Scid^* mice, supporting a diagnosis of AML.

### Leukemic Thymocytes from *IgHμ-TLX1^Tg^Prkdc^Scid/Scid^* mice Exhibit Molecular Features Reminiscent of Human T-ALL

**Figure 6:**
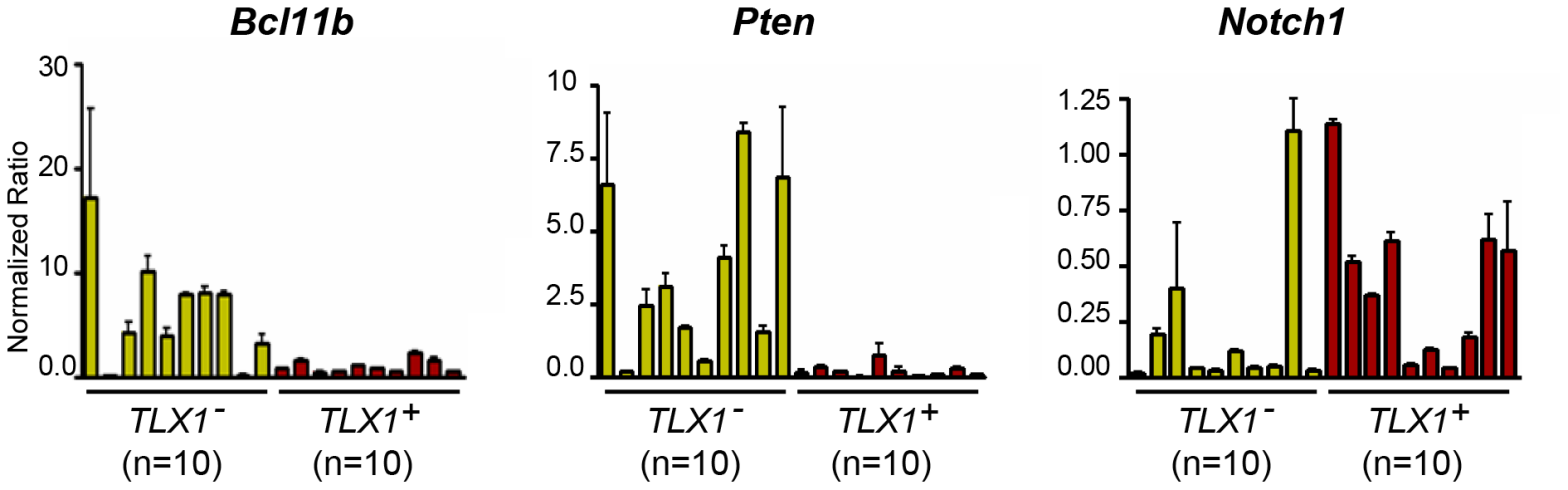
qRT-PCR analysis of thymic tumors derived from *Prkdc^Scid/Scid^* and *IgHμ-TLX1^Tg^Prkdc^Scid/Scid^* mice. qRT-PCR analysis of expression of *Bcl11b*, *Pten* and *Notch1* in tumors isolated from ten *Prkdc^Scid/Scid^* and ten *IgHµ-TLX1^Tg^Prkdc^Scid/Scid^* mice. Data were normalized relative to *β-actin*. Each bar represents one tumor sample.

To test whether the T-ALL-like disease seen in the *IgHμ-TLX1^Tg^Prkdc^Scid/Scid^* mice resembled human T-ALL at a molecular level, we assessed the expression of several genes which had previously been shown to be associated with the progression of human T-ALL such as *NOTCH1*, *PTEN* and *BCL11B* [5], [13], [29], [30] in ten tumors isolated from *IgHμ-TLX1^Tg^Prkdc^Scid/Scid^* and *Prkdc^Scid/Scid^* mice. Consistent with gene expression patterns in human T-ALLs, these analyses revealed a statistically significant (p<0.05) reduction in the expression of *Bcl11b* and *Pten* in T-ALL arising in *IgHμ-TLX1^Tg^Prkdc^Scid/Scid^* mice relative to tumors isolated from *Prkdc^Scid/Scid^* mice (Figure 6). Furthermore, elevated expression of *Notch1* was detected in six of ten tumors isolated from *IgHμ-TLX1^Tg^Prkdc^Scid/Scid^* mice relative to *Prkdc^Scid/Scid^* mice (p<0.05). These data indicated that malignant thymocytes derived from *IgHμ-TLX1^Tg^Prkdc^Scid/Scid^* mice exhibited a similar pattern of expression of a subset of genes that has previously been implicated in *TLX1*-initiated human T-ALL.

### Gene Expression Profiling in Premalignant Thymocytes Reveals Dysregulated Expression of Genes Involved in Cell Cycle, Apoptosis and Chromosome Segregation

**Figure 7:**
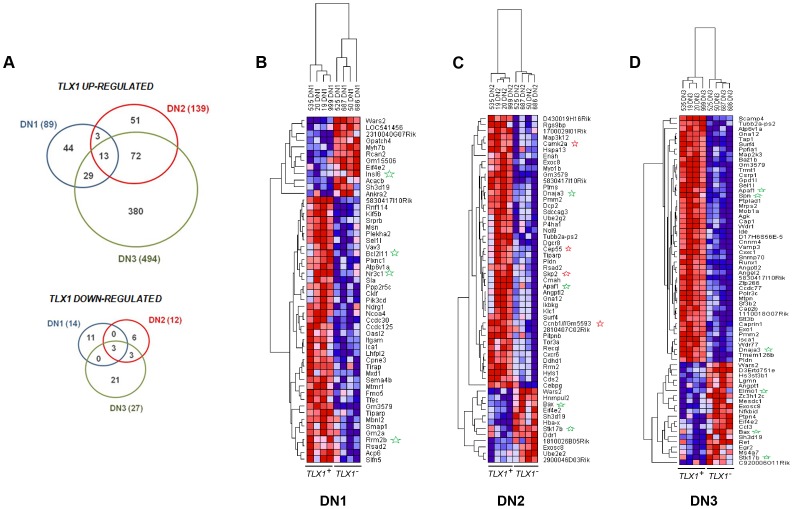
Heat map of the top ranking differentially expressed genes in flow sorted premalignant thymocytes. (A) Venn diagrams depicting TLX1-associated up-regulated and down-regulated genes in DN1, DN2 and DN3 fractions. (B–D) Gene expression heat map of the top ranking differentially expressed genes in DN1 (B), DN2 (C) and DN3 (D) flow-sorted thymocytes from *IgHµ-TLX1^Tg^Prkdc^Scid/Scid^* and *Prkdc^Scid/Scid^* mice. For each heat map, the first four columns (*TLX1* ^+^) show *IgHµ-TLX1^Tg^Prkdc^Scid/Scid^* thymocyte-derived samples, and the last four columns (*TLX1* ^-^) represent samples derived from thymocytes of *Prkdc^Scid/Scid^* mice.

Next, we sought to interrogate the molecular pathways dysregulated in the earliest stages of *TLX1*-induced transformation in the context of *Prkdc* deficiency. Therefore, we purified premalignant DN1, DN2 and DN3 thyomocytes from four cohorts of *IgHμ-TLX1^Tg^Prkdc^Scid/Scid^* mice and four cohorts of control *Prkdc^Scid/Scid^* mice, and compared their gene expression profiles using Affymetrix Gene Chip microarrays (Figure S1A–B). Each cohort consisted of 2–5 sex- and age-matched mice. From this analysis, we identified 103 *TLX1*-associated differentially expressed genes (DEGs) in DN1 thymocytes, (89 up regulated genes, 14 down regulated genes), 151 DEGs in DN2 thymocytes (139 up regulated, 12 genes down regulated) and 522 DEGs in DN3 thymocytes (495 up regulated, 27 genes down regulated) (Figure 7A, Tables S1–3). The over-abundance of DEGs in the DN3 fraction suggested that the effects of TLX1 on target gene expression were more profound at more mature stages of thymocyte development.

Heat maps of the top ranked DEGs for each DN fraction are shown in Figure 7B–D. Analysis using the Database for Annotation, Visualization and Integrated Discovery (DAVID) software revealed a disproportionate representation of cell cycle- and apoptosis-related genes amongst the TLX1-associated DEGs across all DN subpopulations. In total, the expression of 4, 12 and 28 genes which regulate cell cycle progression, and 10, 15 and 48 genes which regulate apoptosis were dysregulated in *IgHμ-TLX1^Tg^Prkdc^Scid/Scid^* DN1, DN2 and DN3 thymocytes, respectively (Tables S1–3). Amongst apoptotic regulators which were dysregulated, the majority were anti-apoptotic genes which were up-regulated (*Prnp, Stambp, Men1, Dhcr24, Aars, Akt1, Bre, Rasa1, Adora2a, Api5, Dnajc5, Stat5a, Tnfaip3, Gsk3b, Ikk-β*, *Pik3cd, Prkacb* and *Akt/Pkb*), while some apoptosis-inducing genes were down regulated (*Bax, Stk17b* and *Elmo1*). Among the differentially expressed cell cycle regulators, genes involved in mitotic spindle checkpoint regulation, such as components of the anaphase promoting complex (APC) (*Anapc1, Anapc5, Cdc23*), regulators of chromosome segregation (*Anapc1, Anapc5, Cdc23, Smc1a, Rad21* and *Stag1)* and cohesion subunits (*Smc1a, Rad21* and *Stag1*) were prominent. Consistent with this, gene set enrichment analysis (GSEA) applied to the DN1, DN2 and DN3 datasets separately also revealed enrichment of genes encoding components of the spindle complex in TLX1-associated targets from DN2 thymocytes (NES = 2.21, q-value = 0.02) (Figure S2).

**Figure 8:**
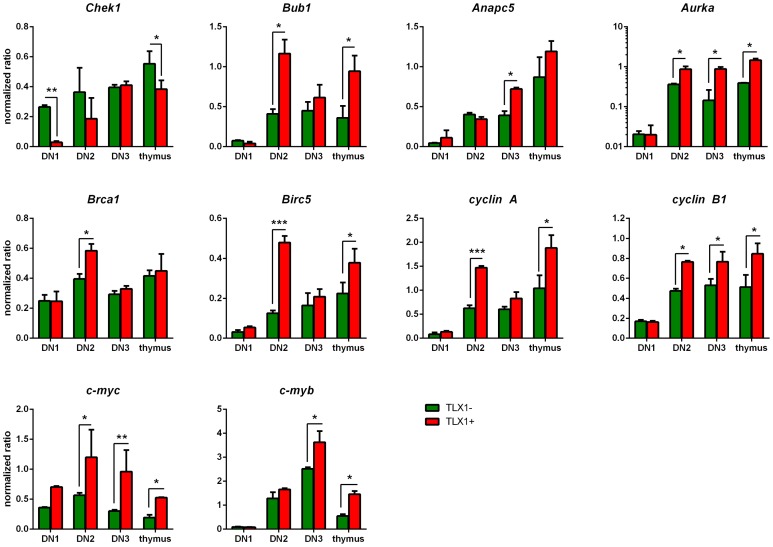
Validation of differential gene expression in premalignant thymocytes *of Prkdc^Scid/Scid^* and *IgHμ-TLX1^Tg^Prkdc^Scid/Scid^* mice. qRT-PCR analysis of selected genes whose protein products are involved in chromosome segregation (*Chek1, Aurka* and *Bub1*), cell cycle progression (*Cyclin A, Cyclin B1, Anapc5, c-myc* and *c-myb*) and apoptosis (*Birc5, Brca1*). Samples are presented as pairs with the first bar representing the level of expression of cells isolated from *Prkdc^Scid/Scid^* mice and the second from *IgHµ-TLX1* ^Tg^ *Prkdc^Scid/Scid^* mice. Data were normalized relative to *β-actin*. Red bars: DN1; brown: DN2; green: DN3 cells; black; total unsorted thymocytes. Error bars represent SD. Statistical testing was performed using the student’s T-test. Statistically significant differences are indicated by asterisks (* depicts p<0.05, ** depicts p<0.01, *** depicts p<0.001).

We next validated dysregulated expression of a subset of DEGs in thymocytes from *IgHμ-TLX1^Tg^Prkdc^Scid/Scid^* and *Prkdc^Scid/Scid^* control mice by qRT-PCR (Figure 8). Analysis of DN1, DN2 and DN3 thymocytes confirmed elevated expression of *Aurka, Bub1* and *Anaps5* in *TLX1*-expressing thymocytes. Furthermore, we demonstrate increased expression of the apoptosis genes, *Brca1* and *Birc5* in DN2 thymocytes and decreased expression of *Chek1* in DN1 thymocytes from *IgHμ-TLX1^Tg^Prkdc^Scid/Scid^* mice. Finally, expression of additional genes involved in cell cycle regulation known to be associated with human T-ALL initiation and progression were also assessed. qRT-PCR analysis confirmed upregulated expression of the cell cycle regulators, *cyclin B1* and *cyclin A* and upregulated expression of the oncogenes, *c-myc* and *c-myb,* in *IgHμ-TLX1^Tg^Prkdc^Scid/Scid^* thymocytes relative to *Prkdc^Scid/Scid^* thymocytes. Combined, these results suggest that *TLX1* in collaboration with loss of DNA-PK dysregulates the expression of genes involved in the regulation of the cell cycle, the spindle checkpoint and chromosome segregation.

### Increased Cellularity of Thymi from *IgHμ-TLX1^Tg^Prkdc^Scid/Scid^* Mice

**Figure 9:**
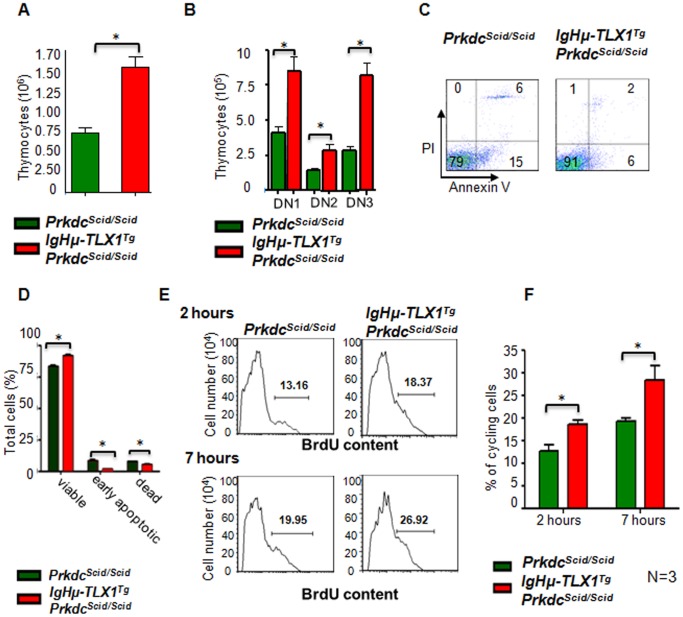
Expression of *TLX1* in *IgHµ-TLX1^Tg^Prkdc^Scid/Scid^* premalignant thymocytes increases cell viability and provides a proliferative advantage. (A) Absolute cell numbers of thymocytes isolated from 20, six week old *IgHµ-TLX1^Tg^Prkdc^Scid/Scid^* and 20 *Prkdc^Scid/Scid^* littermates (p<0.0001). (B) Thymocytes were flow sorted based on expression of CD44 and CD25 and absolute numbers of DN thymocytes were calculated using percentages obtained after flow sorting (p<0.0001). (C) Thymocytes from three, six week old *IgHµ-TLX1^Tg^Prkdc^Scid/Scid^* and *Prkdc^Scid/Scid^* littermates were stained with Annexin V and PI than assessed by flow cytometric analysis for cell viability. The lower left quadrant of each panel contains viable cells, the upper right quadrant contains dead cells and the lower right quadrant contains early apoptotic cells. The percentage of cells in each quadrant is indicated. (D) Percentages of viable, apoptotic and dead thymocytes in thymi of *IgHµ-TLX1^Tg^Prkdc^Scid/Scid^* and *Prkdc^Scid/Scid^* littermates, as determined by flow cytometry with Annexin V and PI staining. Error bars represent SD. (E) Histogram showing premalignant thymocytes obtained from *Prkdc^Scid/Scid^* and *IgHµ*-*TLX1^Tg^Prkdc^Scid/Scid^* mice, 2 and 7 hours after intraperitoneal injection with 10 µM BrdU. (F) The percentages of proliferating cells in thymi of *IgHµ-TLX1^Tg^Prkdc^Scid/Scid^* and *Prkdc^Scid/Scid^* littermates as determined by BrdU and PI staining. Data represent means of triplicate measurements with error bars to represent ± SD (p<0.0001). Statistically significant differences between compared samples are indicated by asterisks.

We predicted that the effect of TLX1 on cell cycle and apoptotic control may cause aberrant thymocyte proliferation. Therefore, to explore the effects of *TLX1* expression on thymus cellularity, assessments of absolute numbers, apoptosis and proliferation of thymocytes were performed using thymi isolated from premalignant *IgHμ-TLX1^Tg^Prkdc^Scid/Scid^* and *Prkdc^Scid/Scid^* mice. Cell counts revealed a statistically significant increase in the absolute number of thymocytes isolated from thymi of *IgHμ-TLX1^Tg^Prkdc^Scid/Scid^* mice relative to *Prkdc^Scid/Scid^* control littermates (Figure 9A). This increased cellularity was observed in all three DN fractions (Figure 9B). Cell viability, as assessed by Annexin V and Propidium Iodide (PI) staining (Figure 9C), revealed an increase in the proportion of viable thymocytes and a reduction in apoptotic and dead thymocytes obtained from *IgHμ-TLX1^Tg^Prkdc^Scid/Scid^* thymi relative to *Prkdc^Scid/Scid^* controls (Figure 9D). Moreover, the proportion of proliferating thymocytes from *IgHμ-TLX1^Tg^Prkdc^Scid/Scid^* mice was also significantly increased, as established by *in vivo* BrdU incorporation (Figure 9E–F). Combined, these data suggested that the increased cellularity of thymi of premalignant *IgHμ-TLX1^Tg^Prkdc^Scid/Scid^* mice results from decreased cell death and increased proliferation of immature DNA-PK-deficient *TLX1*-expressing thymocytes.

### Aneuploidy and Spindle Checkpoint Dysfunction in Premalignant Thymocytes and Thymic Tumors

**Figure 10:**
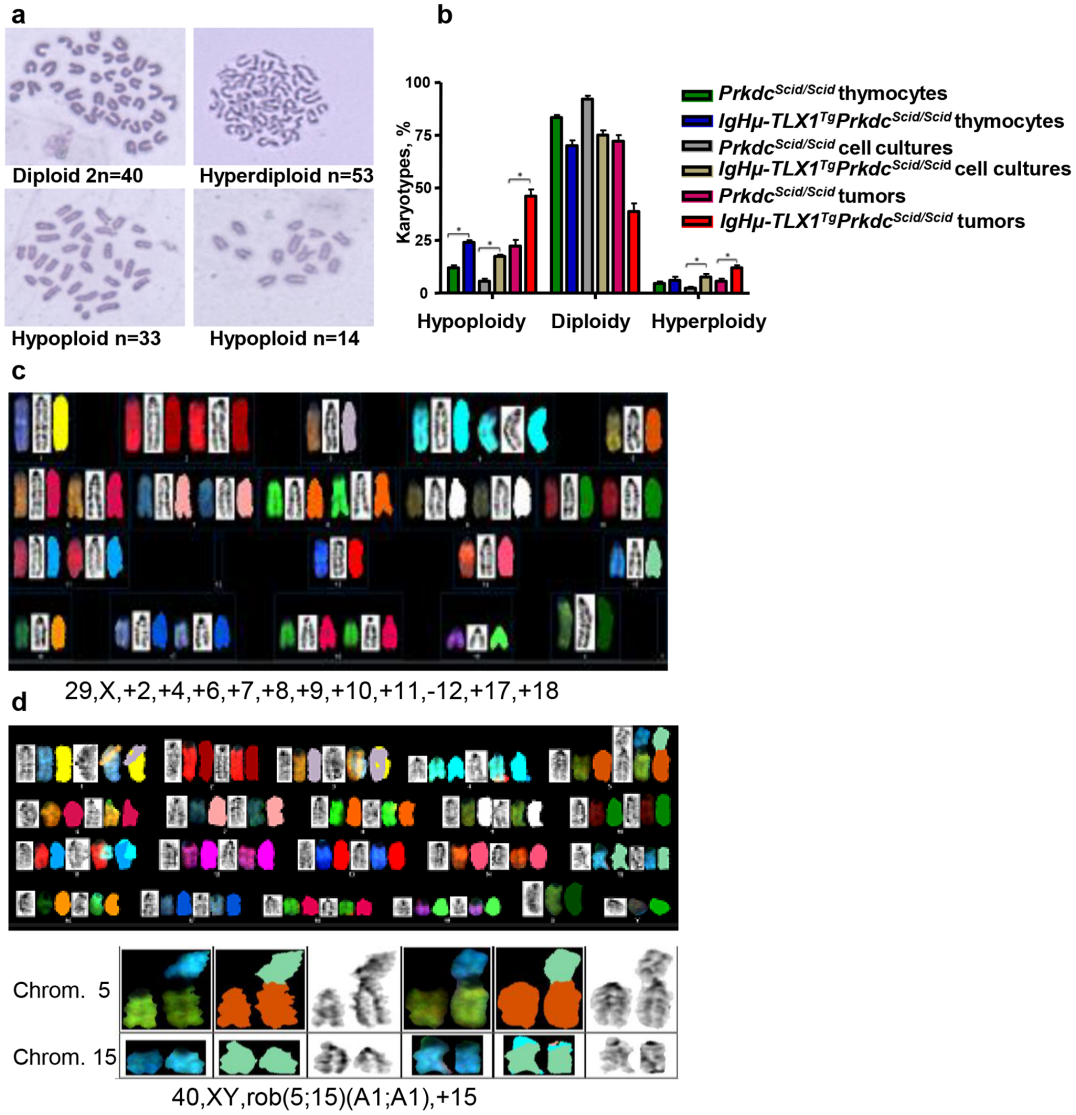
Chromosome analysis of premalignant thymocytes and tumors from *Prkdc^Scid/Scid^* and *IgHµ-TLX1^Tg^Prkdc^Scid/Scid^* mice. (A) Representative micrographs of Giemsa stained diploid, hyperdiploid, and hypoploid chromosome spreads from *IgHµ-TLX1^Tg^Prkdc^Scid/Scid^* mice. (B) Ploidy assessment of cultured thymocytes and thymic tumors obtained from premalignant and moribund *Prkdc^Scid/Scid^* and *IgHµ-TLX1^Tg^Prkdc^Scid/Scid^* mice, respectively. For each of three samples, 40 to 100 metaphase spreads were analyzed. The percentage of aneuploid spreads relative to the total number of analyzed spreads was determined. Statistically significant differences (p<0.05) are indicated by asterisks. (C) Spectral karyotype analysis of a hypoploid tumor isolated from an *IgHµ-TLX1^Tg^Prkdc^Scid/Scid^* mouse with T-ALL. Loss of chromosome 12 and gain of chromosome 17 were found in 10% and 5% of analyzed cells, respectively. The karyotype is indicated below. (D) An abnormal, unbalanced trisomy 15 karyotype with a rob(5;15) translocation and two normal copies of chromosome 15. Examples of the rob(5;15) chromosomes and chromosome 15 are shown. The karyotype is indicated below.

Previous studies revealed an association of *TLX1* overexpression with dysregulated chromosome segregation [5], [17]. To determine whether a similar phenomenon was also a feature of malignant *TLX1^Tg^Prkdc^Scid/Scid^*-derived T cells, we compared chromosome numbers in metaphase spreads prepared from thymocytes and tumor cells obtained from premalignant and terminally ill *IgHμ-TLX1^Tg^Prkdc^Scid/Scid^* and *Prkdc^Scid/Scid^* mice. Premalignant thymocytes were analyzed directly after isolation of thymi and after *in vitro* culture in the OP9-DL1 co-culture system. These analyses indicated that premalignant thymocytes and tumor cells of *IgHμ-TLX1^Tg^Prkdc^Scid/Scid^* mice had a statistically significant increase in the numbers of aneuploid cells relative to *Prkdc^Scid/Scid^* samples (Figure 10A–B).

Spectral karyotype (SKY) analysis of three tumors isolated from *IgHμ-TLX1^Tg^Prkdc^Scid/Scid^* mice confirmed these results. In addition, these studies revealed a recurrent loss of chromosome 12 and gain of 17 in the cells of two independently arising tumors (Figure 10C). Moreover, two of three tumors showed an unbalanced trisomy 15 with tumor cells containing two normal chromosome 15 and a Robertsonian translocation consisting of chromosome 5 and chromosome 15, replacing one chromosome 5. Breakpoints in both the 5 and 15 chromosomes were located within the centromeric region (Figure 10D).

**Figure 11:**
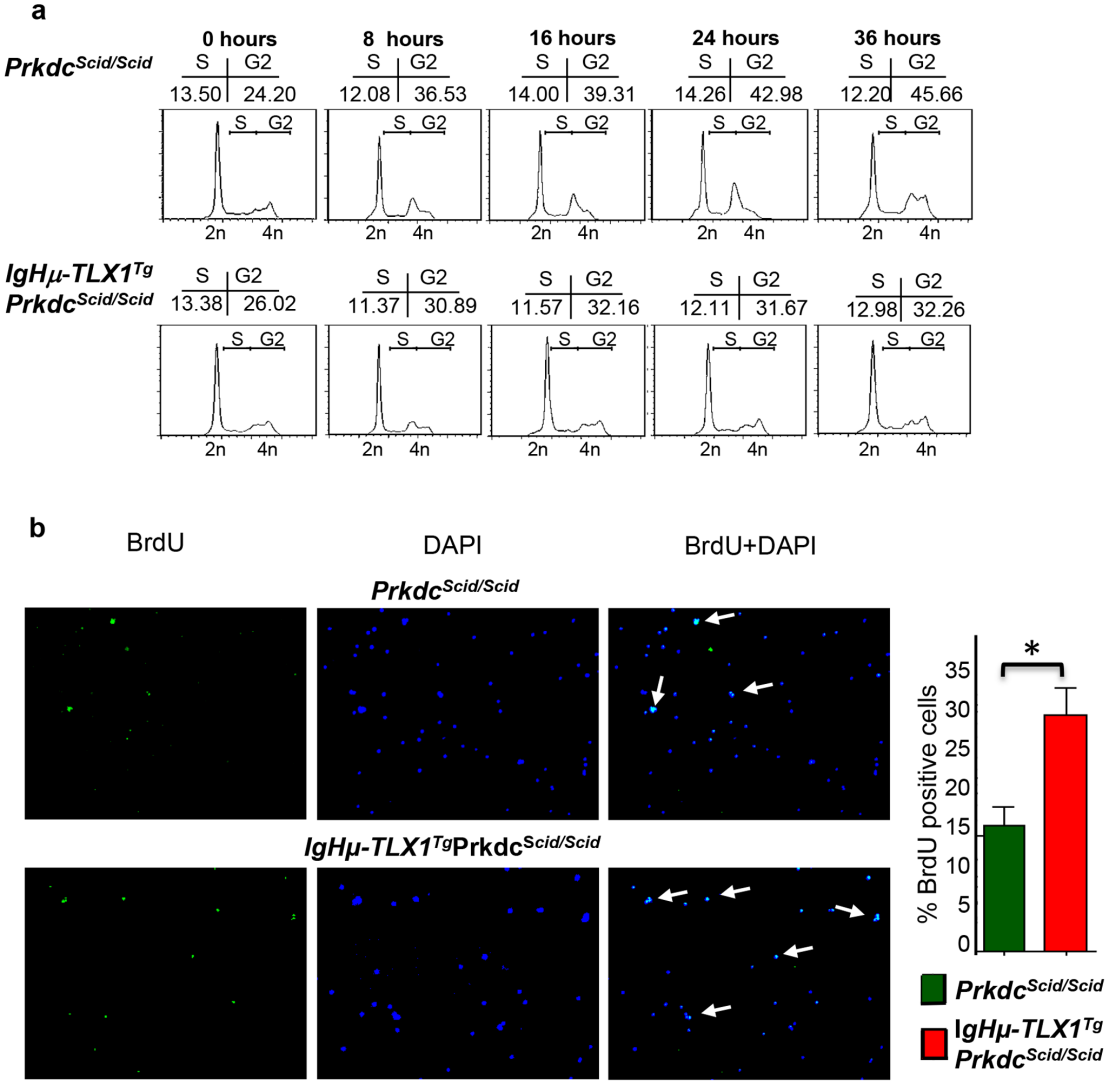
Aberrant checkpoint regulation in thymocytes of *IgHµ-TLX1^Tg^Prkdc^Scid/Scid^* mice. (A) Cell cycle analysis showing the percentage of thymocytes in S and G2/M as determined by PI staining for DNA content. Percentages of cells in S and G2/M are shown above the histograms. (B) BrdU labeling to assess bypass of the G2/M cell cycle checkpoint in *Prkdc^Scid/Scid^* and *IgHµ-TLX1^Tg^Prkdc^Scid/Scid^* thymocyte cultures. Thymocytes were treated with colchicine to induce mitotic arrest then exposed to BrdU to assess cell cycling. BrdU incorporation was detected by BrdU immunolabeling and nulcei were revealed by DAPI staining. White arrows on the merged images indicate cycling thymocytes. The histogram depicts the mean percentages of BrdU-positive cells assessed by scoring 20 random fields. Statistically significant differences (p<0.05) are indicated by asterisks.

We speculated that the abnormal karyotypes detected in thymocytes of *IgHμ-TLX1^Tg^Prkdc^Scid/Scid^* mice were due to dysregulation of the spindle cell cycle checkpoint. To address this possibility, we compared the frequency at which premalignant thymocytes from *IgHμ-TLX1^Tg^Prkdc^Scid/Scid^* and *Prkdc^Scid/Scid^* mice bypassed the spindle cell cycle checkpoint. Thymocytes derived using the OP9-DL1 co-culture system were arrested with colchicine, a mitotic spindle poison, and the proportion of cells in G1, S or G2/M phase of the cycle determined at eight-hour intervals by analysis of DNA content. In two independently performed experiments, *IgHμ-TLX1^Tg^Prkdc^Scid/Scid^* thymocytes showed a reduced proportion of thymocytes in G2/M at all-time points assessed, relative to *Prkdc^Scid/Scid^* thymocytes, suggesting an increased ability to bypass this checkpoint (Figure 11A). To further verify the ability of *IgHμ-TLX1^Tg^Prkdc^Scid/Scid^* premalignant thymocytes to bypass the spindle cell cycle checkpoint, cells were subjected to colchicine-induced spindle checkpoint arrest and then cultured in the presence of BrdU. Incorporation of BrdU into the nucleus was used as an indicator of cell division. These analyses indicated a statistically significant increase in BrdU positive thymocytes for *IgHμ-TLX1^Tg^Prkdc^Scid/Scid^* mice relative to thymocytes from *Prkdc^Scid/Scid^* mice (30% ±4.6 versus 16.3% ±5.1; p<0.05) (Figure 11B). Taken together, these results indicated that the aneuploidy observed in *Igμ-TLX1^Tg^Prkdc^Scid/Scid^* thymocytes resulted from dysregulated control of the spindle checkpoint in *TLX1* overexpressing cells.

## Discussion

The first transgenic mouse model confirming the oncogenic potential of *TLX1 in vivo* was reported by Hough *et al* [11]. These mice expressed elevated levels of *TLX1* throughout all stages of B cell development, during the early stages of thymocyte development and in myeloid progenitors. A premalignant stage involving mature B cells was detected in mice as early as eight weeks of age. Most mice died of a mature B cell lymphoma although ∼20% died of myeloid hyperplasia. The average age of death was 14 months. More recently, two transgenic mouse models were described in which ectopic *TLX1* expression was restricted to the T cell compartment. These mice develop a T-ALL-like disease with a disease latency of 35 weeks months [5], [12] In both reports, there was an extended latency prior to the development of malignant disease, suggesting that ectopic expression of *TLX1*, by itself, was insufficient to initiate malignancy and that additional mutations were required for premalignant cells to progress to full malignancy.

In this report, we shed light on an additional process, namely the loss of the DNA repair enzyme DNA-PK, which collaborates with *TLX1* in neoplastic transformation. *IgHμ-TLX1^Tg^Prkdc^Scid/Scid^* mice rapidly died of T-ALL or AML at a significantly reduced latency compared to *Prkdc^Scid/Scid^* control mice. That a proportion of mice developed myeloid malignancies was consistent with our previous report that expression of *TLX1* in myeloid progenitors predisposed mice to myeloid hyperplasia [11]. Furthermore, these studies indicated that the dysregulated expression of *TLX1* did not influence disease phenotype, but rather, accelerated malignant transformation of not only T-ALL, but also AML in *IgHμ-TLX1^Tg^Prkdc^Scid/Scid^* mice.

Our report aso provides some insights into possible molecular mechanisms by which DNA-PK insufficiency synergizes with *TLX1* expression to accelerate T-ALL in mice. Given its role as a key DNA repair enzyme in the NHEJ pathway, we were somewhat surprised that malignant thymocytes from *IgHμ-TLX1^Tg^Prkdc^Scid/Scid^* mice did not exhibit increased incidences of translocations, amplifications or end-to-end telomere fusions. Rather, these cells instead exhibited increased incidences of aneuploidy. It remains unclear whether this reflects a direct or indirect effect of DNA-PK insufficiency. Of note, in addition to its canonical role in the NHEJ pathway, DNA-PK has also been implicated as a regulator of chromosome segregation [22], [23], and its loss in *IgHμ-TLX1^Tg^Prkdc^Scid/Scid^* mice may directly compromise spindle checkpoint function. Alternatively, the aneuploidy in *IgHμ-TLX1^Tg^Prkdc^Scid/Scid^* mice may reflect indirect consequences of gene expression changes, such as attenuated expression of key spindle checkpoint proteins. Similar to our findings, thymocytes from a p56*^Lck^-TLX1* mice model also exhibit attenuated *Chek1* expression, which is associated with spindle checkpoint dysfunction and a predisposition towards aneuoploidization [5]. Interestingly, in that study, decreased expression of spindle checkpoint factors *Aurka* and *Bub1* was observed, whereas increased expression was noted in the *IgHμ-TLX1^Tg^Prkdc^Scid/Scid^* mice. This discrepancy may be linked to variations in *TLX1* expression in the different mouse models, differences in the genetic backgrounds of the mice and a gene expression modulatory effect of DNA-PK deficiency. For several of these spindle checkpoint proteins, however, both increased and decreased expression can been correlated with checkpoint dysfunction [31], [32].

It is worth noting that, despite vastly different methodological approaches, the karyotypic abnormalities seen in *IgHμ-TLX1^Tg^Prkdc^Scid/Scid^* mice (+13, +17, -12) recapitulated those reported in other murine models of T-ALL [5]. It is likely that these chromosomes harbor oncogenes and tumor suppressors that may be especially relevant in T-ALL pathogenesis. For example, the putative tumor suppressor gene *Bcl11b* resides on mouse chromosome 12 and *BCL11B* is deleted in ∼16% of human T-ALL [5]. These overlaps indicate that the pathways which serve to maintain proper ploidy is particularly important in T-ALL biology, and may be a frequent node for disruption for the nascent T-cell malignancy. While, to our knowledge, mutations in the *Prkdc* gene have not been identified in whole exome sequencing efforts of two cohorts of primary T-ALL patients [33], [34], further data mining and sequencing efforts to identify lesions in other components of the NHEJ pathway as well as other DNA repair pathways in T-ALL may be warranted.

## Materials and Methods

### Mice

Mice were bred and maintained under pathogen-free conditions and monitored daily for signs of illness. Mice were euthanized and necropsied when moribund. All manipulations were performed under Canadian Animal Care protocols and approved by the Sunnybrook Research Animal Care Committee (Protocol number: 12-005). Double mutant *IgHμ-TLX1^Tg^Prkdc^Scid/Scid^* mice were generated by mating previously described *IgHμ-TLX1^Tg^* mice (original name *IgHμ-HOX11* transgenic mice [12], [13]), bred onto the CD1 genetic background, with CB17 ICR- *Prkdc^Scid/Scid^* mice bred onto an ICR genetic background (Charles River Laboratories (Wilmington, MA, USA). *IgHμ-TLX1^Tg^Prkdc^Scid/Scid^* and *Prkdc^Scid/Scid^* mice were mated and progeny were genotyped and used to generate cohorts.

The presence of the *IgHμ-TLX1* transgene was revealed by PCR amplification of a 287-bp fragment using 5′-AACCGCAGATACACAAAGGA-3′ forward and 5′-TGGGCCAGGCTCTTCTGGAA-3′ reverse primers. Mice homozygous for the *Prkdc* mutation (*Prkdc^Scid/Scid^*) were detected by flow cytometry analysis of peripheral blood stained with pan B-cell specific (B220 clone RA3-6B2, 1∶200 dilution) and pan T-cell specific (CD3 clone 145-2C11, dilution 1∶100) antibodies.

### Flow Cytometry

Single cell suspensions of murine thymi, spleens and bone marrow were prepared in Dulbecco’s phosphate-buffered saline (D-PBS) (BD Pharmingen) containing 0.5% BSA (FACS buffer). Fc receptor blocking was performed by preincubation with an unconjugated anti-Fcγ antibody (clone 2.4G2; 1 mg/ml 1∶500 for thymocytes, 1∶200 for splenocytes and bone marrow cells) for 10 minutes on ice. 10^6^ cells were then stained with cocktails of fluorochrome-conjugated antibodies appropriately diluted in FACS buffer for 30 minutes at 4°C then washed twice in 5 ml of FACS buffer. Fluorescein isothiocyanate (FITC), R-phycoerythrin (PE) or allophycocyonin (APC)-conjugated antibodies and dilutions used were: CD25-APC (Clone PC61.5; 1∶300), CD44-PE (Clone IM7; 1∶200), CD4-PE-Cy7 (Clone RM4-5; 1∶200), CD8-APC-Cy7 (Clone 53-6.7; 1∶200), CD3-PerCP-Cy5 (Clone 145-2C11; 1∶100), TCR-β-Alexa Fluor 700 (Clone H57-597; 1∶100), c-kit-APC (CD117) (Clone 2B8; 1∶800), Sca1-PE (Clone E13-161; 1∶100) (all from BD Pharmingen, Franklin Lakes, NJ, USA), Thy1.2-PE (Clone 30-H12 1:400), TCR-αβ-FITC (Clone H57; 1∶200), B220-PE (Clone RA3-6B2; 1∶200), Gr-1-FITC (Clone RB6-8C5; 1∶100), Mac-1-APC (Clone M1/70; 1∶200), CD4-APC (Clone GK1 1:800), CD8-FITC (Clone 53-6.7.2; 1∶400) (all from the Sunnybrook Core Hybridoma Facility, Toronto, ON, Canada). Cells were resuspended in 300 µl FACS buffer containing 1 µg/ml PI (Invitrogen, Carlsbad, CA, USA) for dead cell exclusion. Samples were analyzed on a FACS Calibur cytometer using CellQuest Pro software (BD Biosciences).

### Reverse Transcriptase-PCR (RT-PCR) and Quantitative RT-PCR (qRT-PCR)

Genomic DNA was purified using DNAzol (Invitrogen) and total RNA was isolated using an RNeasy kit (Qiagen, Valencia, CA, USA). Total RNA was amplified with MessageAmp™ II aRNA kit (Invitrogen) according to the manufacturer specifications. The *IgHμ-TLX1* transgene was detected with the following primers: h*TLX1*-1560F (5′-TGGGCATCTATGGGAGAGTG-3′) and h*TLX1*-1799R (5′-CTGGATTGGGCTGGGATGT-3′). The following PCR conditions were used: initial denaturation at 95°C for 3 minutes; 34 cycles of denaturation (95°C) for 30 seconds, annealing (55°C) for 30 seconds, extension (72°C) for 30 seconds and a final cycle of 7 minutes at 72°C. All reactions were carried out in a 25 µl volume containing 50–100 ng genomic DNA for PCR or 10–50 ng total RNA for reverse transcribed cDNA, and 0.2 µmol of each primer, 10x buffer, 1.5 U AmpliTaq DNA Polymerase and 2.5 µM MgCl_2_ (Invitrogen) using the PCR C1000 Thermal Cycler (BioRad). The identity of PCR products was verified by DNA sequencing (The Center for Applied Genomics, The Hospital for Sick Children, Toronto, ON).

First-strand cDNA synthesis was performed using the SuperScript III First Strand cDNA reverse transcriptase kit and random primers (Invitrogen). qRT-PCR was performed with the QuantiTect SYBR Green PCR kit (Qiagen) according to the manufacturer’s protocol with 1 µl of the reverse transcription reaction product (from 10–50 ng total RNA) amplified for 39 cycles with an ABI PRISM 7000 Applied Biosystems Sequence Detection System and analyzed with ABI Prism 7000 SDS software (Applied Biosystems, Carlsbad, CA, USA). qRT-PCR primers are listed in Table 2.

**Table 2:** List of primers.

primer	F	R
*hTLX1*	5′-TGGGCATCTATGGGAGAGTG-3′	5′-CTGGATTGGGCTGGGATGT-3′
*mBcl2*	5′-GCGTCAACAGGGAGATGTCA-3′	5′-TCAAACAGAGGTCGCATGCT-3′
*mAnapc5*	5′-GAGTCGCTGAATGCGGGT-3′	5′-TGGGCGTGCTGACTGTTG-3′
*mPten*	5′-GGCACAAGAGGCCCTAGATTT-3′	5′-CGGGTCTGTAATCCAGGTGA-3′
*mChek1*	5′-TGGGAATTTGGTGCAAACTTT-3′	5′-CAGGGTGAGGGTCAGATGG-3′
*mBcl11b*	5′-CAGGGTGAGGGTCAGATGG-3′	5′-GAACCAGGCGCTGTTGAAG-3′
*mBrca1*	5′-TTGCCTGCCAAGGCGAGA-3′	5′-TGCCAATGTGGGCTGGCT-3′
*mAurka*	5-CAGAAGAATGAGCAGCCTGCA-3′	5′-CTGTTCCAAGGGGCGCATAT-3′
*mNotch1*	5′-CGCCCTTGCTCTGCCTAAC-3′	5′-CACTTCGCACCTACCTCCATT-3′
*mCyclin A*	5-GTCACCACATACTATGGACATG-3′	5′-AAGTTTTCCTCTCAGCACTG-3′
*mc-myb*	5′-CCTTCTCTCCCTCGCAGTT-3′	5′-TGCTCTCAAGGCAGAAACTG-3′
*mc-myc*	5′-GCTCTGCTGTTGCTGGTGATA-3′	5′-GCTCTGCTGTTGCTGGTGAT-3′
*mBirc5*	5′-ATCCACTGCCCTACCGAGAA-3′	5′-TCCATCTGCTTCTTGACAGTG-3′
*mCyclinB1*	5′-TCAGGGTCACTAGGAACACGA-3′	5′-GCAAGTTCCACCTCTGGTTC-3′
*mBub1*	5′-CATGAGCAGTGGGTTAGTGAA-3′	5′-TTCTCAGAAGCAGGAAGGTC-3′
*mβ-actin*	5′-AGAGGGAAATCGTGCGTGAC-3′	5′-AGGTCTTTACGGATGTCAAC-3′

### *In vivo* Thymocyte Proliferation Assay

Healthy *IgHµ-TLX1^Tg^Prkdc^Scid/Scid^* and age and sex matched *Prkdc^Scid/Scid^* littermates were injected intraperitoneally with 150 µl of 10 mg/ml BrdU in sterile 1X D-PBS. Mice were sacrificed 2 and 7 hours post injection, thymi were harvested and single cell suspensions prepared in D-PBS containing 2% FBS. Cells were fixed in 80% ethanol for 30 minutes and washed in 1X D-PBS for 5 minutes. Cells were then treated with RNase (Invitrogen) and 1.5 M HCl for 30 minutes. 5×10^5^–10^6^ pretreated cells were stained with an anti-BrdU mouse monoclonal antibodies (1∶100, Trevigen, Gaithesburg, MD) for 30 minutes at 4°C, washed in 5 ml of 1X D-PBS and stained with a FITC-conjugated anti-mouse secondary antibody (1∶500, BD Pharmingen). After three washes, cells were resuspended in 300 µl 1X D-PBS and analyzed using a FACS Calibur cytometer and CellQuest Pro software.

### Co-Culture of Thymocytes on OP9-DL1 Stromal Cells

GFP-expressing OP9-DL1 cells (kindly provided by Dr. Juan Carlos Zúñiga-Pflücker) were cultured in 6-well dishes (BD Falcon) to 80% confluence in αMEM (Gibco) medium containing 20% FBS (Hyclone) and 100 U/ml Penicillin/Streptomycin (Gibco). Cultures were incubated in a humidified atmosphere at 37°C with 5% CO_2_. Fetal livers were dissected from E13.5 embryos and dispersed to single cell suspensions in HBSS supplemented with 10% FBS by passage through a 40 µm nylon cell strainer (BD Falcon). Ten percent of cells were used for genotyping with the remaining cells depleted of lineage-restricted cells using biotin-labeled antibodies specific for mouse lineage specific antigens (B220, CD3ε, Gr-1, Mac-1, Ter119 and F4/80; BD Pharmingen). Lineage-positive cells were bound to Miltenyi Biotec streptavidin magnetic beads and depleted using a MACS column according to manufacturer’s instructions (Miltenyi Biotech, Auburn, CA USA). Lineage negative (Lin^-^) HSCs were sorted for expression of the CD117/c-kit (APC) and Sca-1 (PE) antigens on a FACS ARIA flow sorter using II FACS DIVA software (BD Biosciences). C-kit^+^Sca^+^Lin^-^ cells (10^3^) were resuspended in 3 ml of complete DMEM medium supplemented with murine Flt-3 Ligand (5 µg/ml), IL-7 (5 µg/ml) and SCF (10 µg/ml; all from R&D Systems, Minneapolis MN, USA) and plated onto an OP9-DL1 feeder layer. Cells were harvested on day-7, passed through a 70 µm cell strainer, stained with CD44 (PE) and CD25 (APC) antibodies and thymocyte subpopulations sorted as follows: CD44^+^CD25^-^ (DN1), CD44^+^CD25^+^ (DN2), CD44^-^CD25^+^ (DN3) and CD44^-^CD25^-^. OP9-DL1 cells were distinguished from thymocytes based on GFP expression.

### Bromodeoxyuridine (BrdU) Labeling and Bypass of the Spindle Cell Cycle Checkpoint

To induce mitotic arrest, day-4 *IgHµ-TLX1^Tg^Prkdc^Scid/Scid^* and *Prkdc^Scid/Scid^* c-kit^+^Sca^+^Lin^-^ HSC-derived thymocytes co-cultured on OP9-DL1 were treated with 100 ng/ml colchicine overnight. BrdU was added to a final concentration of 20 µM and incubated at 37°C for 2 hours. T-lymphocytes were collected by centrifugation at 1,300 rpm for 5 minutes and fixed in 80% ethanol, followed by DNA denaturation in 2 N HCl for 1 hour at 37°C. Cells were pretreated by incubation with 1X D-PBS supplemented with 2% BSA for 1 hour at 37°C. Immunostaining was performed using an anti-BrdU antibody for 2 hours at RT, followed by staining with a FITC-conjugated anti-mouse antibody for 1 hour at RT. Counterstaining for detection of nuclei was performed with VECTASHIELD DAPI mounting medium (Vector Laboratories, Inc. Burlingame, CA). Slides were evaluated using fluorescence microscopy (Axiovert 200 M, Zeizz, GmbH, Germany). In all, 20 random fields were scored for the frequency of BrdU positive cells.

To assess spindle checkpoint bypass following mitotic arrest, day 4 *IgHµ-TLX1^Tg^Prkdc^Scid/Scid^* and *Prkdc^Scid/Scid^* HSC-derived thymocytes, co-cultured in the OP9-DL1 system, were subjected to a double-thymidine block, treated with 100 ng/ml colchicine then harvested at 8 hour intervals, stained with PI and analyzed by flow cytometry for DNA content as previously described [35]. For each sample, 10,000 events were acquired using a FACS Calibur and cell cycle analysis was performed using FlowJo software (BD Biosciences).

### Cytogenetic and Ploidy Analysis of Tumors

Primary leukemic cells were dissected from lymphoid tissues of moribund *IgHµ-TLX1^Tg^Prkdc^Scid/Scid^* mice and immediately processed to single cell suspensions. Fresh tumor samples were placed in RPMI 1640 medium (Wisent; Montreal, Quebec, Canada) containing 10% FBS. Cells were arrested in metaphase by treatment with colcemid (0.03 µg/mL; Invitrogen). Metaphase chromosomes were prepared according to standard cytogenetic protocols, using 0.075 M KCl and Carnoy’s fixative. Slides were aged overnight at 55°C then G-banded using 4X USP Pancreatin (diluted to 0.4X) followed by staining with Leishmann/Giemsa stain. 20 metaphase spreads were analyzed and imaged with a CCD camera (VDS Vosskühler GmbH) using an Olympus microscope set in bright-field mode with a 100X/N.A. 1.40 oil immersion objective. Co-ordinates were recorded for metaphase relocation following Spectral karyotyping (SKY). Prior to SKY, slides were destained with Carnoy’s fixative, rehydrated with an ethanol series, pretreated with 2X SSC at 37°C, then post-fixed with 1% formaldehyde/PBS/MgCl_2_ and washed in PBS. SkyPaint™ probes were used according to the manufacturer’s instructions (Applied Spectral Imaging, Carlsbad, CA). Metaphases were viewed with an Olympus BX61 microscope (Olympus, Center Valley, USA) equipped with a SpectraCube SD300 (Applied Spectral Imaging, Migdal HaEmek, Israel). SKY images were analyzed using SkyView Version 2.1.1 (Applied Spectral Imaging).

Abnormalities in chromosome number of mitotic cells were estimated by calculating the proportion of aneuploid thymocytes and tumor cells relative to cells displaying a normal karyotype. 40 and 100 random metaphases of thymocytes and tumor cells, respectively, were photographed and chromosomes were enumerated using light microscopy (LEICA DM LB2, Bannockburn, IL, USA). Metaphase chromosome spreads were prepared using standard Geimsa staining techniques.

### Histology and Immunohistochemistry

Organs isolated from moribund *IgHµ-TLX1^Tg^Prkdc^Scid/Scid^* and *Prkdc^Scid/Scid^* mice as well as healthy *IgHµ-TLX1^Tg^Prkdc^Scid/Scid^* mice were fixed in 4% PFA overnight, paraffin embedded, sectioned and stained with hematoxylin-eosin (Sigma, St. Louis, MO, USA). A certified pathologist performed all morphological interpretations of tissue sections. Tumors were also cryopreserved in O.C.T. compound (Tissue-Tek; Fisher Scientific, Waltham, MA, USA) for cryosectioning and 5 µm sections were stained with purified rat anti-mouse Thy1.2 (clone 53-2.1; BD Pharmingen). Antibody binding was visualized using an anti-rat IgG-HRP (Santa Cruz Biotechnology) and Peroxidase Substrate Kit DAB (Vector Laboratories, Inc.). Sections were counterstained with hematoxylin 1∶5 (Fluka).

### Statistics

Disease-free survival during a 25-month observation period was calculated from the date of birth to the date when mice became terminally ill using the Kaplan-Meier estimator. Column comparison analysis was performed using the logrank test with the GraphPad Prism 4 software. Student’s t-test was used to detect differences in cycling thymocytes between *IgHµ-TLX1^Tg^Prkdc^Scid/Scid^* and *Prkdc^Scid/Scid^* cultures. All tests with p<0.05 were accepted as statistically significant.

### Sample Isolation and High-density Oligonucleotide Microarray Screening

Double mutant *IgHµ-TLX1^Tg^Prkdc^Scid/Scid^* and control *Prkdc^Scid/Scid^* mice were sacrificed at 4–8 weeks of age. DN1, DN2, DN3 and CD44^-^CD25^-^ subpopulations were sorted based on expression of CD44 and CD25. To minimize sample variability caused by individual differences among animals, individual thymocyte subpopulations from sex-matched mice were pooled (total 10^4^–10^5^ cells). Each pool consisted of between 2–5 age- and sex-matched *IgHµ-TLX1^Tg^Prkdc^Scid/Scid^* or control *Prkdc^Scid/Scid^* mice. The pooled cell pellets were homogenized in TRIzol reagent (Invitrogen) and stored at -80°C for total RNA isolation. Total RNA was isolated and purified with RNeasy kits according to the manufacturer’s instructions. *TLX1* genotypes were confirmed prior to microarray experiments. Microarray processing was performed on the Affymetric platform according to manufacturer’s protocols.

Raw microarray CEL data files were imported to GeneSpring GX11 (Silicon Genetics, Redwood City, CA). Data was subjected to background reduction, filtered for genes flagged as Present or Marginal in at least 12.5% of samples, prior to standardization by normalization to the mean of all samples. Statistically significant differentially expressed genes (DEGs) were determined by a two-tailed, unpaired t-test, with DEGs defined as those with a p≤0.05 and a minimum average fold change of 1.4. Gene annotation queries were conducted through Affymetrix NetAffx Analysis Center, and the Database for Annotation, Visualization and Integrated Discovery (DAVID). Gene Set Enrichment Analysis (GSEA) was performed to analyze enrichment of gene sets following the developer’s protocol (http://www.broadinstitute.org/gsea/index.jsp). Analysis was done using 1,000 gene set permutations.

### Accession Code

Microarray data are available in the Gene Expression Omnibus (GEO) under accession number GSE47421.

## Supporting Information

Figure S1 **Methodology for isolation of DN fractions for gene expression profiling.** (A) Schematic representation of work flow for isolating purified DN1, DN2 and DN3 fraction from pools of *Prkdc^Scid/Scid^* and *IgHµ-TLX1^Tg^Prkdc^Scid/Scid^* mice for gene expression profiling. (B) Characteristics of the 8 mouse cohorts analyzed in this study.
(PDF)

Figure S2 **GSEA analysis shows positive enrichment for spindle components in** ***IgHµ-TLX1^Tg^Prkdc^Scid/Scid^*** **premalignant thymocytes.** (A) Enrichment plots showing up-regulation of mitotic spindle and tubular formation genes in DN2 thymocytes from *IgHµ-TLX1^Tg^Prkdc^Scid/Scid^* mice. (B) Heat map depicting expression patterns of mitotic spindle and tubular formation genes in DN2 fractions of *Prkdc^Scid/Scid^* and *IgHµ-TLX1^Tg^Prkdc^Scid/Scid^* mice. Red and blue indicate higher and lower expression, respectively.
(PDF)

Table S1 **List of differentially expressed genes in** ***IgHμ-HOX11^Tg^Prkdc^Scid/Scid^*** **DN1 cells.**
(XLSX)

Table S2 **List of differentially expressed genes in** ***IgHμ-HOX11^Tg^Prkdc^Scid/Scid^*** **DN2 cells.**
(XLSX)

Table S3 **List of differentially expressed genes in** ***IgHμ-HOX11^Tg^Prkdc^Scid/Scid^*** **DN3 cells.**
(XLSX)
