# Supplementary material for: Ectopic TLX1 Expression Accelerates Malignancies in Mice Deficient in DNA-PK
Source: PLoS One. 2014 Feb 26;9(2):e89649. doi: 10.1371/journal.pone.0089649 (PMC3935916; doi:10.1371/journal.pone.0089649)
Supplement: Figure S1 — Methodology for isolation of DN fractions for gene expression profiling. (A) Schematic representation of work flow for isolating purified DN1, DN2 and DN3 fraction from pools of PrkdcScid/Scid and IgHµ-TLX1TgPrkdcScid/Scid mice for gene expression profiling. (B) Characteristics of the 8 mouse cohorts analyzed in this study. (PDF) [file pone.0089649.s001.pdf]

A

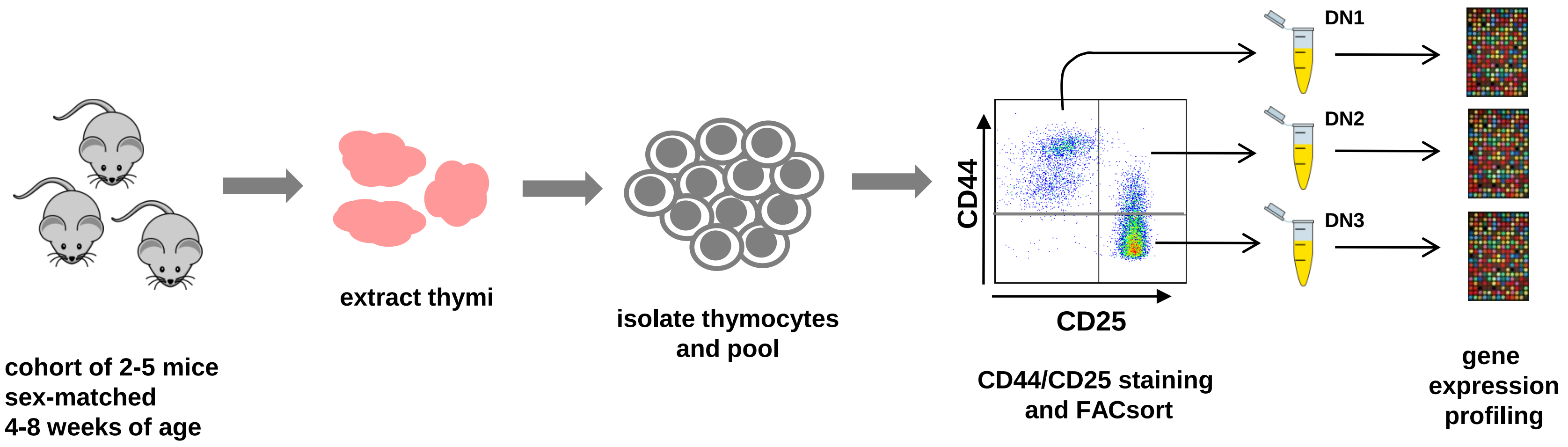

B

Cohort Descriptor	Genotype	Sex	# Mice in cohort	Age (weeks)	% DN1	% DN2	% DN3	% non-DN1,2,3
999	HOX11-SCID	M	2	6	31.0%	28.6%	39.6%	0.9%
19	HOX11-SCID	F	5	4-6	27.0%	35.9%	37.0%	0.1%
535	HOX11-SCID	F	4	7	36.3%	15.5%	48.0%	0.2%
20	HOX11-SCID	M	3	4-7	40.1%	18.8%	19.9%	21.2%
525	SCID	F	5	7-8	32.9%	38.5%	28.6%	0.1%
687	SCID	M	2	6-7	37.5%	26.0%	36.3%	0.2%
50	SCID	F	2	4-6	26.8%	39.9%	33.0%	0.2%
686	SCID	M	2	6	21.9%	4.9%	7.8%	65.4%

Figure S1.
