# Supplementary figures and images for: Ectopic TLX1 Expression Accelerates Malignancies in Mice Deficient in DNA-PK
Source: PLoS One. 2014 Feb 26;9(2):e89649. doi: 10.1371/journal.pone.0089649 (PMC3935916; doi:10.1371/journal.pone.0089649)

A

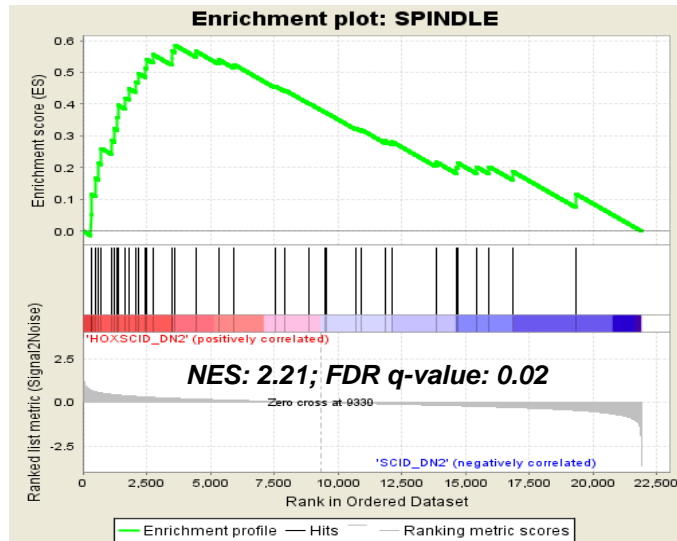

B

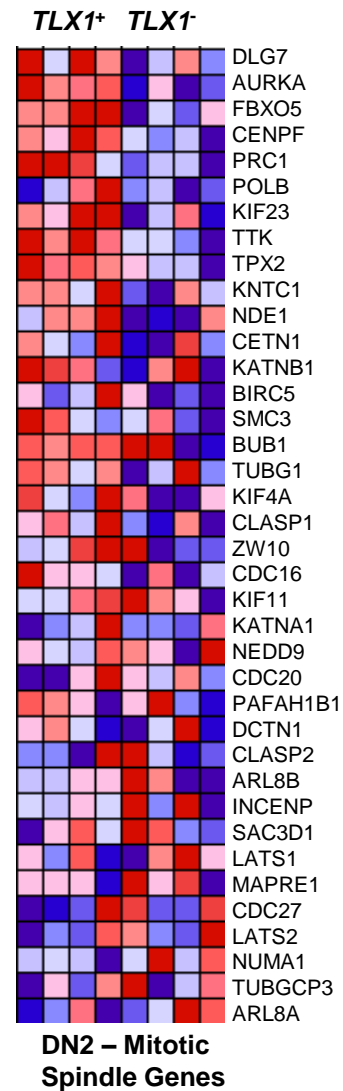

Figure S2.

Supplement: Figure S2 — GSEA analysis shows positive enrichment for spindle components in IgHµ-TLX1TgPrkdcScid/Scid premalignant thymocytes. (A) Enrichment plots showing up-regulation of mitotic spindle and tubular formation genes in DN2 thymocytes from IgHµ-TLX1TgPrkdcScid/Scid mice. (B) Heat map depicting expression patterns of mitotic spindle and tubular formation genes in DN2 fractions of PrkdcScid/Scid and IgHµ-TLX1TgPrkdcScid/Scid mice. Red and blue indicate higher and lower expression, respectively. (PDF) [file pone.0089649.s002.pdf]
